# Oral probiotic administration attenuates postexercise inflammation in horses

**DOI:** 10.1093/tas/txae124

**Published:** 2024-08-13

**Authors:** Robert D Jacobs, Daniel Grum, Benjamin Trible, Diana I Ayala, Theodore P Karnezos, Mary E Gordon

**Affiliations:** Land O Lakes, Purina Animal Nutrition, Gray Summit, MO 63039, USA; Land O Lakes, Purina Animal Nutrition, Gray Summit, MO 63039, USA; Land O Lakes, Purina Animal Nutrition, Gray Summit, MO 63039, USA; Land O Lakes, Purina Animal Nutrition, Gray Summit, MO 63039, USA; Land O Lakes, Purina Animal Nutrition, Gray Summit, MO 63039, USA; Land O Lakes, Purina Animal Nutrition, Gray Summit, MO 63039, USA

**Keywords:** equine, exercise, inflammation, nutrition, probiotic

## Abstract

Probiotics are commonly incorporated into equine diets to impart health and performance benefits; however, peer-reviewed evidence supporting their efficacy in horses is limited. Interestingly, bacteria from the *Bacillus* genus are gaining interest for their unique ability to impact metabolic, immune, and inflammatory pathways. The objective of this trial was to evaluate a selection of *Bacilli* for their role in altering the inflammatory response in horses to exercise. Eighteen horses were utilized in a randomized cross-over trial. Horses were randomly assigned to one of 6 starting treatments including a negative and positive control, and groups that received one of 4 probiotics (*Bacillus coagulans* GBI-30, 6086, *Bacillus subtilis*-1, *Bacillus subtilis*-2, or *Bacillus amyloliquefaciens*) top dressed to their daily ration at a rate of 8 billion CFU/d mixed into dried whey powder. All horses received a similar base diet of grass hay offered at 2.0% of bodyweight daily along with 4.54 kg of a commercially available textured horse feed. Each 3-wk phase of the trial consisted of a 2-wk dietary acclimation followed by a 1-wk exercise challenge and sample collection. Between phases, horses were offered only their base diet. On the day of exercise, horses were offered their 0700 ration and then subjected to a 2-h standardized exercise test. Blood samples were obtained prior to starting exercise and then again at 0, 2, 4, 6, 8, 24, 48, and 72-h postexercise. Horses in the positive control group were administered 0.23 mg/kg BW flunixin meglumine immediately following the 0-h sampling. Samples were analyzed for serum amyloid A (SAA), interleukin-6 (IL-6), and prostaglandin E_2_ (PGE_2_) concentrations. Data were evaluated via ANOVA using the MIXED procedure in SAS 9.4. Exercise-induced inflammation as evidenced by SAA, IL-6, and PGE_2_ increases postexercise. Horses consuming *B. coagulans* GBI-30, 6086 had reduced production of SAA, IL-6, and PGE_2_ compared to all other probiotic-fed groups and the negative control (*P* < 0.001). The positive control successfully ameliorated the postexercise inflammatory response. These data highlight the potential for *B. coagulans* GBI-30, 6086 to be incorporated into equine rations as a method to support optimal response to exercise or other inflammation-inducing challenges. Additional research is ongoing to elucidate the methodology by which these results occur.

## Teaser

Supplementation of *Bacillus coagulans* GBI-30, 6086 reduces exercise-induced inflammation and supports horses through the exercise recovery period.

## Introduction

The use of probiotics to impact animal health and performance has long been established (Schoster et al., 2014). However, the majority of studies in horses have focused on the ability of these additives to impact the hindgut fermentation process (Costa and Weese, 2012; Costa et al., 2012). Recently, researchers have noted the ability of these unique bacteria to impart benefits to host health and performance through a variety of pathways including immune modulation (Guarner and Malagelada, 2003; Jones and Versalovic, 2009), antimicrobial production (Saarela et al., 2000), competitive exclusion of pathogenic bacteria (Collado et al., 2007), and inhibition or inactivation of bacterial toxins (Chen et al., 2006; Allaart et al., 2011). In fact, the most recent definition proposed by the World Health Organization and the Food and Agricultural Organization specifically mentions the ability of these “live microorganisms to...provide a beneficial effect beyond that of their nutritional value” (Kechagia et al., 2013).

There are a variety of commonly utilized microorganisms in equine rations including but not limited to the following genera: *Saccharomyces* (yeast), *Lactobacillus*, *Bacteroides*, *Enterococcus*, and *Streptococcus* (Dougal et al., 2013). These have been evaluated due to their relatively high abundance in the colon of the horse and evidence of health-promoting benefit in other species. However, the presence in the colon and impact on overall gastrointestinal health have not been definitively linked. As defined, probiotics must remain alive through manufacturing and storage, a consideration evaluated in previous research (Weese and Martin, 2011). Further, most research on probiotic bacteria has relied upon the assumption that they survive and colonize the equine gastrointestinal tract even after cessation of administration (Kauter et al., 2019), a concept which may limit the application of novel probiotic strains. While it is true that in foals, colonization of certain probiotics has been recorded, the microbiome of mature horses is much less plastic and significantly less likely to allow for the establishment of novel bacterial colonies (Weese et al., 2003).

*Bacillus* species are widespread in nature and are found across species commonly isolated from the gastrointestinal tract of animals and present in a wide range of environments (Sorokulova et al., 2008). This genus of bacterium is spore-forming and has demonstrated an ability to survive in situations that would otherwise kill other commonly utilized probiotic genera (Nicholson et al., 2000). The most commonly researched strains of *Bacillus* include *Bacillus subtilis*, *Bacillus coagulans*, *Bacillus pumilus*, and *Bacillus licheniforms* (Bernardeau et al., 2017). Non-equine studies have identified a variety of health benefits related to the administration of *Bacillus* probiotics including but not limited to effects on metabolic function (Yang et al., 2015), antioxidant activity (Paik et al., 2005), antimicrobial ability (Jeon et al., 2017; Ayala et al., 2023), and immune modulation (Sarkar et al., 2016). Because of these wide-ranging and varied effects on health and performance, and the increased robustness of these bacteria, these species are well suited, and of interest, to support equine health and performance.

Inflammation is a necessary response by the immune system to injury, disease, infection, or other cellular signals (Schmid-Schonbein, 2006). In horses, inflammation is necessary for optimal immune response to disease as well as exercise recovery and training. However, excess, or prolonged systemic inflammation may result in decreased performance, increased susceptibility to disease, tissue damage, or chronic inflammatory conditions (Vick et al., 2008). Most commonly, inflammation in horses is treated with anti-inflammatory pharmaceuticals or other modalities such as cryotherapy, massage, ultrasound, or laser therapy. Probiotic administration to attenuate inflammation in horses has not yet been widely investigated.

The objective of this trial was to evaluate the effects of 4 unique strains of *Bacillus* on their ability to alter the inflammatory response of horses undergoing an acute exercise challenge. Based on previous (unpublished) in vitro evaluation of a range of *Bacilli* using tissue culture, it was hypothesized that one or more of the probiotics would impart an anti-inflammatory effect in vivo, demonstrating a potential unique and specific application of probiotic administration to horses.

## Materials and Methods

### Animal Care Statement

This study was conducted at the Purina Animal Nutrition Center in Gray Summit, MO. The animal care and handling procedures used in this study along with the sampling protocols were approved by the Institutional Animal Care and Use Committee (Protocol ID: HR367).

### Animals and Diet

Eighteen unfit horses of Thoroughbred and American Quarter Horse breeding were utilized for this trial. Table 1 outlines the breakdown of age, breed, sex, body weight (BW), and body condition score of the horses. All horses were housed in a single barn, in individual box stalls (12ʹ × 12ʹ) from approximately 1500–0730 hours daily. Daily turnout was provided by treatment group and gender into approximately 1 acre dry-lots from approximately 0730–1500 hours daily. During turnout and while in stalls, horses had free-choice access to clean water and white salt blocks. Horses were offered 4.54 kg daily of a commercially available textured feed (Purina Omolene 500) split into 2 equal meals at 0700 and 1530 hours daily. Additionally, horses were offered 2.0% BW as grass hay at 1530 hours feeding. Following turnout at 0730 hours daily, horses were offered 2.27 kg/head grass hay fed by turnout group. Feed and hay samples were obtained and evaluated for nutrient profile (Equi Analytical, Ithaca, NY). Table 2 provides a detailed analysis of the diet.

**Table 1.** Horse enrollment demographics

American quarter horse (*n*)	10
Thoroughbred (*n*)	8
Mare (*n*)	5
Gelding (*n*)	13
Age (yr ± SEM)	8.4 ± 1.3
Starting BCS (± SEM)	5.2 ± 0.5
Starting BW (kg ± SEM)	511.3 ± 8.5

**Table 2.** Nutritional analysis of diets offered to horses

Nutrient	Grass hay	Purina Omolene 500
DE, Mcal/kg	2.18	3.52
Crude protein, %	9.50	12.60
ADF, %	39.00	13.40
NDF, %	61.70	29.50
Crude fat, %	2.90	8.60
Calcium, %	0.33	0.91
Phosphorus, %	0.20	0.46
Magnesium, %	0.22	0.32
Potassium, %	1.63	1.16
Sodium, %	0.02	0.33
Iron, mg/kg	85.00	234.00
Zinc, mg/kg	13.00	227.00
Copper, mg/kg	6.00	59.00

### Experimental Procedure and Sampling

All horses were on a similar diet prior to enrollment in the trial. Prior to dietary transition a baseline blood sample was obtained via jugular venipuncture and collected in vacutainer tubes (K2 EDTA and SST, Beckton Dickson, NJ) for separation of plasma and serum and analyzed for a complete blood count and for common serum chemistries. All horses were within normal reference ranges for all measured parameters. Horses were transitioned to experimental diets over the course of 2 d. All horses were randomly assigned to one of 6 starting dietary treatment groups. All horses received 15 g of dried whey powder top dressed on to their daily rations at 0700 and 1530 feedings. Horses in the probiotic groups received 4 billion CFU of one of 4 probiotic bacteria (*Bacillus coagulans* GBI-30, 6086 (Kerry Group, County Kerry, Ireland), *Bacillus subtilis*-1, *Bacillus subtilis*-2, or *Bacillus amyloliquefaciens*) mixed into the dried whey powder. Horses were offered their individual diets for 3-wk periods during which weeks 1–2 were used for dietary acclimation and week 3 was used for exercise challenge and sample collection. Following the final collection on week 3, all horses underwent a 2-d washout and were randomly assigned to a subsequent treatment group. The trial was designed as a randomized cross-over with all horses completing all treatments.

At the start of week 3 of each phase, horses underwent a forced exercise challenge on a circular equine exerciser (Equi-Line Mfg, Burlington, ON). On the morning of the exercise challenge, all horses were offered their 0700 feed approximately 30 min prior to the start of exercise. All horses were exercised for a 2-h period consisting of 4 replications of walk (2.5 m/s) for 3 min, trot (4.5 m/s) for 7 min, and canter (6.2 m/s) for 5 min each in both clockwise and counterclockwise directions. Blood samples were obtained immediately following the cessation of exercise (0-h postexercise) from all horses via jugular venipuncture into vacutainer tubes containing K2 EDTA and blank serum separator tubes for separation of plasma and serum respectively. Additional samples were collected at 2, 4, 6, 8, 24, 48, and 72-h postexercise. Plasma and serum were immediately processed, aliquoted, and stored at −80 °C until analysis. Horses in the positive control group were administered 0.23 mg/kg BW flunixin meglumine (Merck Animal Health, Rahway, NJ) IV immediately following the 0-h postexercise sample collection. Following exercise, all horses were monitored to ensure they returned to resting heart and respiration rates within 60 min postexercise. Following the 8-h postexercise sample collection, all horses were bathed to remove excess sweat buildup.

### Inflammatory Marker Analysis

Serum amyloid A (SAA) was evaluated using an automated latex bead-based immunoturbidometric assay (SAA-Vet, Eiken Chemical Co, Japan) at the Cornell Animal Health Diagnostic Center (Ithaca, NY). Prostaglandin E_2_ (PGE_2_) was determined in blood samples using a commercially available kit according to manufacturer’s instructions (Enzo Life Science, Farmingdale, NY). The intra- and inter-assay CV for PGE_2_ analysis were 8.6% and 13.2%, respectively. Interleukin-6 (IL-6) was determined in blood samples using a commercially available kit according to manufacturer’s instructions (R&D Systems, Minneapolis, MN). The intra- and inter-assay CV for IL-6 analysis were 7.2% and 11.8%, respectively.

### Statistical Analysis for Inflammatory Markers

Inflammatory marker data were analyzed via ANOVA using the MIXED procedure of SAS 9.4 (SAS Inst. Inc., Cary, NC) with horse as the experimental unit. The model included the fixed effect of timepoint and treatment as well as their interactions. An α of 0.05 or less determined significance with tendencies considered between 0.05 and 0.10. Data are presented as mean ± SEM.

## Results

### Inflammatory Markers

**Figure 1.**
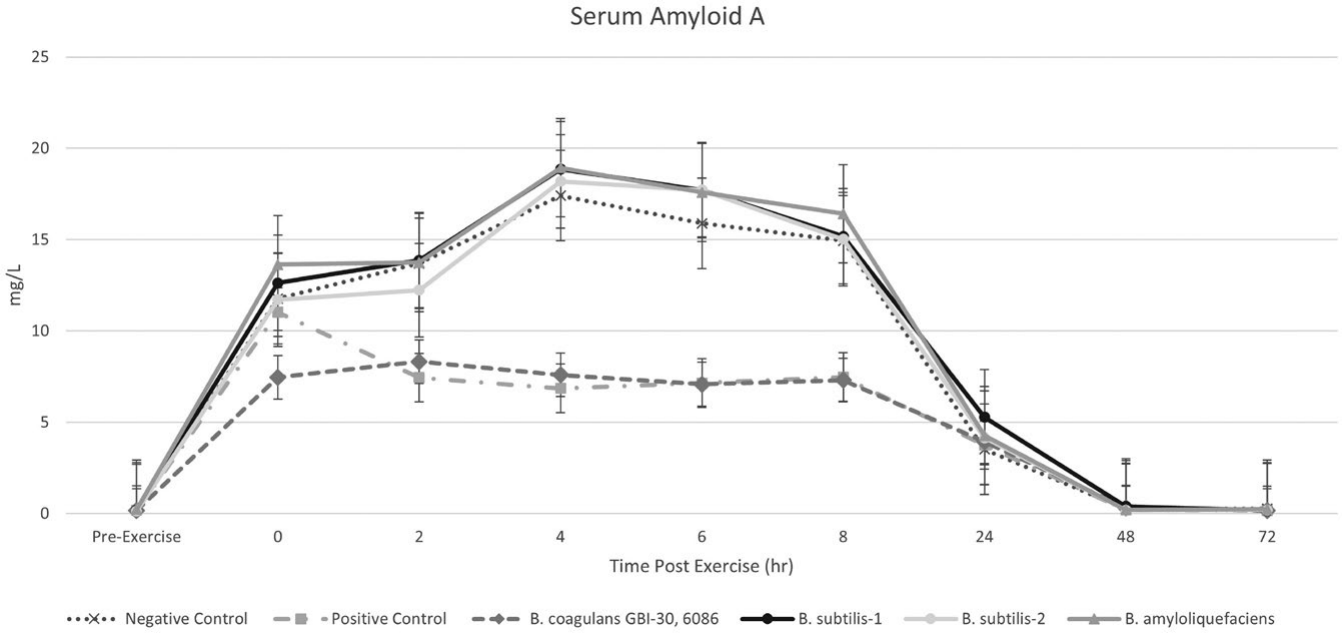
Serum amyloid A levels in horses following an exercise challenge.

**Figure 2.**
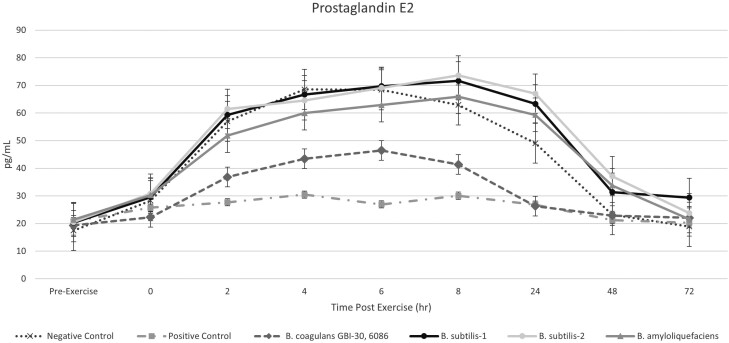
Prostaglandin E_2_ levels in horses following an exercise challenge.

**Figure 3.**
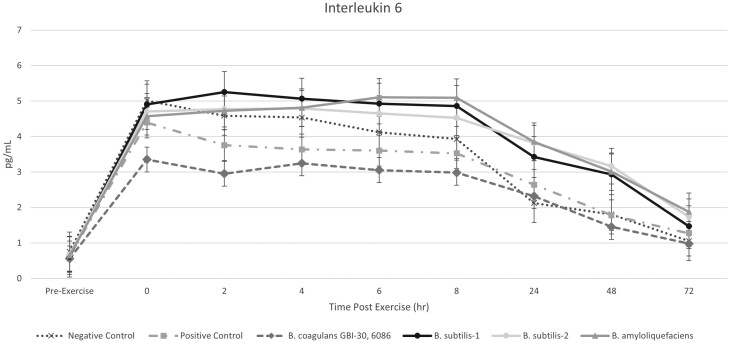
Interleukin-6 levels in horses following an exercise challenge.

Utilization of exercise to induce inflammation was successful for all measured variables (Figures 1–3). Serum amyloid A levels increased immediately postexercise, compared to pre-exercise levels, for all treatment groups (*P* < 0.05). The *B. coagulans* GBI-30, 6086 group had a reduced SAA level at the 0-h postexercise timepoint compared to all other treatment groups (*P* < 0.001). At the 2-h postexercise timepoint, the positive control group had similar SAA levels as the *B. coagulans* GBI-30, 6086 group, and both remained lower compared to all other treatment groups (*Bacillus subtilis*-1, *Bacillus subtilis*-2, or *Bacillus amyloliquefaciens*) until the 24-h postexercise sampling timepoint (*P* < 0.001). Serum Amyloid A expression in the negative control and all other probiotic-supplemented groups was similar (*P* > 0.05) to each other through the duration of the sampling period (Figure 1).

Interleukin-6 followed a similar expression pattern to that of SAA with a rapid increase at 0-h postexercise (*P < *0.001). However, horses supplemented with *B. coagulans* GBI-30, 6086 had an overall reduced expression of IL-6 compared to all other treatment groups (*P* < 0.001). Administration of flunixin meglumine in the positive control group provided a quicker reduction in IL-6 compared to all other probiotic treatments except for *B. coagulans* GBI-30, 6086 (*P* < 0.001). Horses in the negative control group had a reduced expression of IL-6 compared to the *B. subtilis*-1, *B. amyloliquefaciens*, and *B. subtilis*-2 groups at the 2-h postexercise timepoint (*P* < 0.05), but levels were similar to the positive control and *B. coagulans* GBI-30, 6086 groups. All treatment groups returned to baseline levels of IL-6 by the 72-h postexercise sampling timepoint (Figure 3).

Prostaglandin E_2_ levels were similar across all treatment groups at the 0-h postexercise sampling timepoint (*P* > 0.05). Beginning at 2-h postexercise, the *B. coagulans* GBI-30, 6086 group had a reduced PGE_2_ expression compared to all other probiotic-administered groups (*P* < 0.001). In addition, PGE_2_ levels returned to baseline quicker when horses were supplemented with *B. coagulans* GBI-30, 6086 compared to all other probiotic treatments (*P* < 0.001). Regardless of treatment, PGE_2_ levels returned to baseline by the 48-h postexercise sampling timepoint (Figure 2). The positive control, flunixin meglumine administration, effectively negated the PGE_2_ expression following administration (*P* < 0.001).

## Discussion

Probiotic application to equine diets and rations is common to help support digestive function and immune health. However, the efficacy of these probiotics, and evidence of their benefits to support their widespread application is lacking (Cooke et al., 2021). Research into the role of probiotics in equine nutrition has been limited, focusing on modulation of the equine microbiome (Dougal et al., 2013), direct impact on the fiber fermentation in the hindgut of the horse (Jouany et al., 2008, 2009), and recently the reduction in pathogenic bacteria (Schoster, 2018). Horse owners and veterinarians are routinely looking for ways to support optimal health and performance for horses, and research into probiotic administration to horses for these purposes is necessary (Tanabe et al., 2014).

*Bacillus coagulans* strains, also referred to as *Weizmannia coagulans*, and previously, *Lactobacillus sporogenes* are increasingly used as probiotics in human nutrition. Administration of these bacteria has largely focused on alleviation of irritable bowel syndrome and similar disease states in humans (Hun, 2009; Majeed et al., 2016; Sudha et al., 2018). However, these conditions are largely characterized by increased inflammation and immune dysfunction. *Bacillus coagulans* GBI-30, 6086 is a unique strain of *B. coagulans* that has demonstrated significant effects on exercise recovery (Jager et al., 2016) and immune function (Baron, 2009). Both of these benefits are supported by the results of this trial.

This trial utilized an exercise model to induce a systemic inflammatory response in horses. Based on the markers measured in this trial, all horses responded appropriately to exercise and displayed an increased level of systemic inflammation. Serum amyloid A (SAA) is an acute-phase protein synthesized in the hepatocyte of the horse (Eckersall and Bell, 2010), and is considered the only major positive acute-phase protein, meaning that its concentrations are typically undetectable in normal horses, with significant increases in horses undergoing an inflammatory challenge (Crisman et al., 2008). Several studies have evaluated the effects of exercise on SAA levels in horses and have found that regardless of fitness level, an increase in SAA was observed in exercised horses (Cywinska et al., 2010, 2013). The postexercise increase in SAA observed in this trial supports previous findings and highlights the utility of this marker for research trials such as this one. As intended, the use of flunixin meglumine as a positive control in this trial was successful, when evaluating the SAA response to its administration. Horses in the positive control group displayed similar 0-h postexercise SAA concentrations to those in the negative control group but experienced a quick reduction in SAA levels following administration of the anti-inflammatory. Previous research evaluating the use of flunixin meglumine postsurgery supports these findings (Busk et al., 2010). The treatment group that received the *Bacillus coagulans* GBI-30, 6086 had a reduced SAA response to exercise at all timepoints. This comparative reduction even at the 0-h postexercise timepoint immediately following exercise is likely due to the fact that the horses had been supplemented with *Bacillus coagulans* GBI-30, 6086 for 14 d prior to the initiation of the exercise trial. This finding is significant in that the application of this probiotic to attenuate the inflammatory response to exercise could prove beneficial to performance horses undergoing long-term or acute training.

It has been reported that in horses, exercise induces a systemic pro-inflammatory response that is highlighted by marked increases in circulating levels of interleukin-6 (Donovan et al., 2007a, 2007b). Similarly to the results for SAA, this trial demonstrates that the ability of *B. coagulans* GBI-30, 6086 to attenuate IL-6-induced inflammation by both: 1) reducing the initial IL-6 production in response to the exercise challenge and 2) hastening the return to baseline levels compared to other treatment groups and the negative control. The ability of probiotics to modulate immune function is a more recent area of research; it is hypothesized that probiotics impart a significant influence on the gut barrier through direct interaction with enterocytes (Azad et al., 2018). Thus, maintenance of optimal enterocyte health in horses is of utmost importance. Increased intestinal permeability is known to lead to disease as the gut barrier is no longer able to protect against the microbial toxins and pathogens present in the gastrointestinal lumen (Stewart et al., 2017). While the specific mode of action by which the *B. coagulans* GBI-30, 6086 reduced IL-6 expression was not elucidated in the current study, further research should be conducted to evaluate the effects of *B. coagulans* GBI-30, 6086 on enterocyte health and intestinal integrity.

Prostaglandin E_2_ is a physiologically active lipid compound that is known to cause pain in horses through activation of nociceptive neurons (Brunner et al., 2020). The administration of nonsteroidal anti-inflammatory drugs such as flunixin meglumine directly reduces the production of PGE_2_, thereby acting as an analgesic and anti-inflammatory. Prostaglandin E_2_ production was increased in response to exercise in this trial, although the lag to peak PGE_2_, was longer than for IL-6 or SAA. Because of this, the positive control group, which received flunixin meglumine immediately postexercise, had almost no expression of PGE_2_, highlighting the success of the positive control in mitigating the inflammatory response. Horses who had been consuming *B. coagulans* GBI-30, 6086 had a reduced expression of PGE_2_, compared to all other probiotic-supplemented groups and the negative control. The levels of PGE_2_, in the *B. coagulans* GBI-30, 6086 supplemented group, were no different than those in the positive control group, indicating that this unique probiotic has an ability to attenuate the inflammatory effects of exercise in a significant manner.

The gastrointestinal tract of the horse is intricately connected to the immune system (Jensen et al., 2010). In fact, approximately 70% of the immune cells are located in the gastrointestinal mucosa and gut associated lymphoid tissue (Ruemmele et al., 2009). Oral probiotics such as *B. coagulans* GBI-30, 6086 are known to influence both the innate and adaptive immune systems through interaction with the various immune cells located in these tissues (Dominguez-Bello and Blaser, 2008). Through these interactions, the immunomodulatory effects of *B. coagulans* GBI-30, 6086 are closely intertwined with cytokine expression and production (Ng et al., 2009). Results from this trial support this interaction and demonstrate a novel application for this probiotic in equine diets.

## Conclusion

The results of this trial indicate that horses supplemented with 8 billion CFU/d of *B. coagulans* GBI-30, 6086 displayed a significantly reduced inflammatory response to exercise. The reduction in overall expression of SAA, IL-6, and PGE_2_ highlights the unique ability of this bacterial strain to reduce the inflammatory load on the horse, thereby promoting a more optimal response to exercise-induced inflammation. Further investigation is warranted to determine the distinct mode of action by which this occurs. These data indicate that inclusion of this specific probiotic bacteria into a daily equine ration may prove beneficial to performance horses or those horses experiencing an increased level of systemic inflammation.
